# From qualitative data to quantitative models: analysis of the phage shock protein stress response in Escherichia coli

**DOI:** 10.1186/1752-0509-5-69

**Published:** 2011-05-12

**Authors:** Tina Toni, Goran Jovanovic, Maxime Huvet, Martin Buck, Michael PH Stumpf

**Affiliations:** 1 Division of Molecular Biosciences, Imperial College London, South Kensington, London SW7 2AZ, UK; 2 Centre for Integrative Systems Biology, Imperial College London, South Kensington, London SW7 2AZ, UK; 3 Division of Biology, Imperial College London, South Kensington, London SW7 2AZ, UK; 4 Department of Biological Engineering, Massachusetts Institute of Technology, Room 32-210, Cambridge, MA 02139 USA

## Abstract

**Background:**

Bacteria have evolved a rich set of mechanisms for sensing and adapting to adverse conditions in their environment. These are crucial for their survival, which requires them to react to extracellular stresses such as heat shock, ethanol treatment or phage infection. Here we focus on studying the phage shock protein (Psp) stress response in *Escherichia coli *induced by a phage infection or other damage to the bacterial membrane. This system has not yet been theoretically modelled or analysed *in silico*.

**Results:**

We develop a model of the Psp response system, and illustrate how such models can be constructed and analyzed in light of available sparse and qualitative information in order to generate novel biological hypotheses about their dynamical behaviour. We analyze this model using tools from Petri-net theory and study its dynamical range that is consistent with currently available knowledge by conditioning model parameters on the available data in an approximate Bayesian computation (ABC) framework. Within this ABC approach we analyze stochastic and deterministic dynamics. This analysis allows us to identify different types of behaviour and these mechanistic insights can in turn be used to design new, more detailed and time-resolved experiments.

**Conclusions:**

We have developed the first mechanistic model of the Psp response in *E. coli*. This model allows us to predict the possible qualitative stochastic and deterministic dynamic behaviours of key molecular players in the stress response. Our inferential approach can be applied to stress response and signalling systems more generally: in the ABC framework we can condition mathematical models on qualitative data in order to delimit e.g. parameter ranges or the qualitative system dynamics in light of available end-point or qualitative information.

## Background

Bacteria have evolved diverse mechanisms for sensing and adapting to adverse conditions in their environment [1,2]. These stress response mechanisms have been extensively studied for decades due to their biomedical importance (e.g. development of antibiotic therapies). With the advent of molecular biology technologies it is now possible to study biochemical and molecular mechanisms underlying stress response signalling. However, due to the complexity of these pathways, the development of theoretical models is important in order to comprehend better the underlying biological mechanisms. Models can be especially useful when a system under study involves a large number of components and is too complex to comprehend intuitively.

Unfortunately, however, suitable models are few and far between. For most systems we lack reliable and useful mechanistic models; this even includes systems that have been attracting considerable attention from biologists and biochemists, and for which substantial amounts of data have been generated. The phage shock protein (Psp) response [3] in bacteria -- in particular in *Escherichia coli *-- is one such system. We know much about the constituent players in this stress response and have a basic understanding of their function and evolution [4]. But so far we lack models that would allow for more detailed quantitative, computational or mathematical analysis of this system.

The Psp system allows *E. coli *to respond to filamentous phage infection and some other adverse extracellular conditions, which can damage the cellular membrane. The stress signal is transduced through conformational changes that alter protein-protein interactions of specific Psp membrane proteins, which mediate the release of a crucial transcription factor. This transcription factor then triggers the transcription of seven *psp *genes that activate and modulate the physiological response to stress, which includes membrane repair, reduced motility and fine-tuning of respiration.

The motivation for the research presented in this manuscript is two-fold: (i) we want to construct and analyze a mechanstic mathematical model for the Psp stress response system; (ii) we will develop and illustrate a general theoretical framework that can be employed to make use of qualitative, semi-quantitative or quantitative data and knowledge about biological systems in order to develop useful explanatory and predictive mathematical models of biological systems.

Our modelling strategy is guided by the following questions: can we reverse-engineer a dynamical model for the Psp response system based on limited qualitative data? How much does this information allow us to delimit the ranges of e.g. kinetic reaction rates of such models? We take a two-step approach: we will first subsume all the available information into a Petri net framework and undertake a structural analysis of the model. We then study the dynamics of the model in stochastic and deterministic frameworks. Since parameter values are unknown, we employ an approximate Bayesian computation (ABC) method based on a sequential Monte Carlo (SMC) framework [5] in order to fit the model to the known facts. This allows us to predict what type of dynamic behaviour we may expect to see in time-course experiments.

As we will show in the context of the Psp response in *E. coli*, such an approach can result in non-trivial insights into the system's dynamics. In particular, we will compare the outputs of analyses assuming stochastic and deterministic models, and show that some aspects of the system, such as qualitative dynamic behaviour and some parameters can already be constrained by using the limited information available. More generally, we will discuss how this procedure can be used in the reverse-engineering of biological systems.

### Introduction to the biology of phage shock protein response

Depending on the type of changing environmental conditions, bacteria can employ different stress response mechanisms. Some of the well studied stress response systems are the RpoE and Cpx extra-cytoplasmic systems [2] and the heat-shock response [6]. The stress response systems that respond to alterations in the bacterial cell envelope are collectively known as extra-cytoplasmic or envelope stress responses. Recently a wealth of information has been obtained about the Psp response system [3,4,7,8], which also belongs to this set of responses.

The Psp response was first observed during the filamentous phage infection of a bacterial cell [9]. It can also be invoked as a result of extreme temperature, osmolarity, mislocalization of envelope proteins called secretins, increase in ethanol concentration and presence of proton ionophores. These conditions damage the bacterial cell envelope, which serves as an ion-permeability barrier for the establishment of the proton motive force (pmf). The pmf is a result of an electrochemical gradient, which is caused by a charge difference due to active pumping of hydrogen ions across the membrane. When the cell envelope is adversely affected, and the Psp response cannot be established, this proton motive force dissipates. A physical change in the membrane and/or an associated biochemical change leads to switching on the cell's stress response. The induction of stress results in increased expression levels of the *psp *genes. The Psp response has been extensively studied in *Escherichia coli *[3,7,8,10-13] and a short overview is given in the following paragraphs.

**Figure 1:**
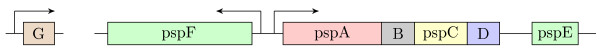
**Genetic arrangement of the PspF regulon**. The regulon consists of the *pspABCDE *operon, *pspF *and *pspG *genes.

The *psp *genes in *E. coli *form the PspF regulon (Figure 1). In *E. coli *the regulon consists of the *psp *operon (containing the *pspA, pspB, pspC, pspD *and *pspE *genes), and the *pspF *and *pspG *genes. PspF is a transcription factor that activates transcription of the *pspA-E *operon, which is driven by a *σ*^54 ^promoter [3,10]. *pspF *is transcribed divergently from the *pspA-E *operon, via a *σ*^70 ^promoter [14]. PspF also activates the transcription of *pspG*.

**Figure 2:**
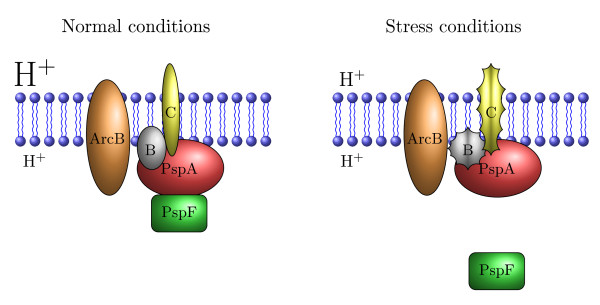
**A schematic model for the Psp response system in E. coli**. A schematic model for the Psp response system in *E. coli*. Under normal conditions, PspA is bound to PspF, which prevents PspF to initiate the transcriptional response. Under stress conditions, PspA and PspF separate in an PspB, PspC and ArcB dependent manner, which allows PspF to initiate the transcription. The sizes of proton symbols H^+ ^around the inner membrane schematically picture the established pmf under normal conditions and dissipated pmf under stress conditions. Under normal conditions the PspA protein plays the role of a *negative regulator*, while under stress conditions PspA turns into an *effector *of the Psp response.

The location of Psp proteins in the cell has been studied in some detail [3,10,12]. PspF is a cytoplasmic protein, PspA is a peripheral inner membrane protein, PspB, PspC, and PspD are inner membrane proteins, PspE is periplasmic, and PspG is an integral inner membrane protein (Figure 2).

Under no-stress conditions the protein PspA binds to PspF, which inhibits the ATPase activity of PspF, resulting in basal transcription of *pspA-E *and *pspG *(Figure 2) [10]. Under Psp inducing conditions (i.e. when stress is present), the stimulus is converted into a signal, which is transduced apparently independently through the transmembrane ArcB sensor kinase, and proteins PspB and PspC. ArcA, a cytosolic cognate response regulator complementing ArcB, plays a role in signal amplification. Through PspB and PspC the signal disrupts the PspA-PspF interaction and allows PspF to activate the transcription. The roles of PspD, PspE and PspG in Psp activation, transduction, transcriptional regulation or membrane repair are not yet fully understood.

The activation of transcription results in the increase in concentration of several Psp proteins. PspA, PspD and PspG play a major role in switching the cell to anaerobic respiration and fermentation, while PspA also binds to the inner membrane phospholipids, repairs the membrane damage and prevents further proton leakage. PspD is also involved in repair of the cell envelope, while PspG play a major role in ne tuning the cell metabolism towards anaerobic respiration and fermentation. Moreover, when over-produced, they all (PspA, D and G) down-regulate cell motility, which in turn down-regulates the pmf consumption and maintains energy usage. Although the PspF regulon and regulation of *psp *genes have been extensively studied, many open questions remain about the kinetics of signal transduction, the function of Psp proteins, and physiological responses. In particular, how does the response evolve over time? How quickly do cells respond to stress when it is induced, and how quickly does the membrane get repaired? Finally, how does the system respond to dissipation or removal of the stress?

Such behaviour is the result of a complex network of interactions, and interplay between the conformational changes of proteins, transcriptional activation and effector activities in the Psp system. All these mechanisms also depend on kinetic rates, which at present are unknown. The system has not yet been theoretically modelled or analysed *in silico*. However, we feel that this rich behaviour cannot be understood using verbal or reductionist models alone. Here we propose to address these questions with the help of mechanistic mathematical models of the system's response. We use inferential techniques to develop mathematical descriptions of a mechanistic model of the Psp response system, analyze these models, and interpret the biological implications of this analysis.

## Results and Discussion

### A mechanistic model of the Psp system

Biological systems are complex and assumptions need to be made and justified whenever building a model to describe their behaviour. It requires biological knowledge, intuition and mathematical skill to develop suitable models that make the right and necessary assumptions in order to simplify the model, while still incorporating all the key players and capturing the necessary level of complexity. Below we first frame our model in the context of a Petri net framework [15-17], which for the present purpose has the benefit of offering a convenient graphical representation that is readily translated into other modelling and simulation schemes. We will make use of some of the specific Petri net tools to check this model, but use ODEs and stochastic processes in order to study the dynamics of these mechanistic models.

In order to build a simple model of the Psp response system, we first need to make some assumptions. In particular, we need to decide which of the molecular species and numerous pieces of biological information have to be included in the Psp model to capture the basic stress response dynamics. Since the proteins PspD, PspE and PspG are only involved in the physiological response and their regulatory role is currently not known, we only include proteins PspA, PspB, PspC and PspF in our simplified model. Moreover, we model proteins PspB and PspC as a complex (*BC*). Proteins ArcA and ArcB play a role in amplifying the signal, but are not necessary for capturing the basic stress response dynamics [8]; we only treat them as an intermediate in passing the signal from the damaged membrane to elicit the change in conformation of PspB and PspC proteins, and will therefore not include them explicitly in the model here.

**Figure 3:**
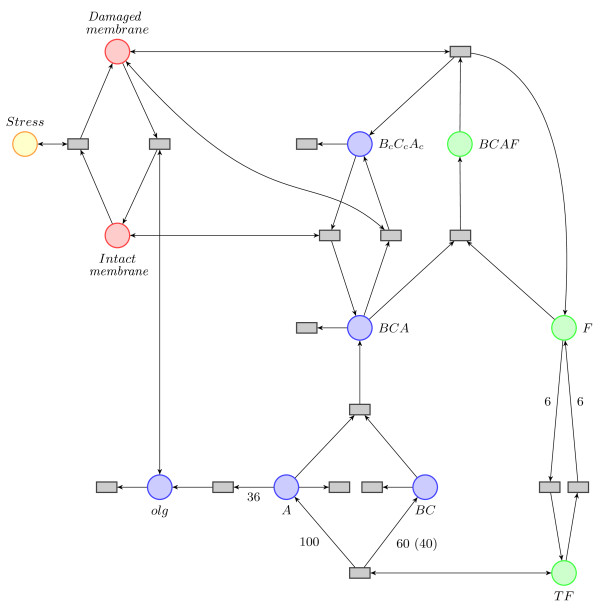
**A Petri net model of the Psp response system**. The starting Petri net model, a graphical representation of reactions (1). The names of places and the model are introduced in the text. Yellow, red and green coloured places correspond to P-invariants (see "Model validation and fitting"), while those coloured in blue correspond to unbounded places.

In the following paragraphs we describe the model in detail (see Figure 3 and reactions 1) and comment on further assumptions that we have made. When the stress acts on the membrane, it inflicts physical damage on it. We measure damage to the membrane in percent (and therefore discretize the membrane so that we can use it in a Petri net framework), and model it as consisting of the "intact membrane" (*im*) parts and the "damaged membrane" (*dm*) parts. When stress acts on the membrane, it can get damaged (eqn. 1a); the proportion of damaged membrane (i.e. the number of tokens in the *dm *place, where the maximum number of tokens is 100) tells us how severely the membrane has been affected.

One of the consequences of membrane damage is dissipation of the proton motive force, which is believed to trigger the conformational changes of proteins PspB, PspC, and presumably PspA as well; in our model this corresponds to complexes *BCA *turning into *B_c_C_c_A_c _*(eqn. 1k). The other consequence of the damaged membrane is that the complex *BCAF *breaks into two parts (eqn. 1i): the first part is PspF, which is then free to form hexamers and acts as a transcription factor (*TF*) (eqn. 1c), and the second part is conformationally changed, *B_c_C_c_A_c_*.

The transcription factor *TF *activates the production of PspA, PspB and PspC proteins (eqn. 1e). The ratio of mRNA production of PspA, PspB and PspC has been experimentally measured as 100:60:40 [7]. Because we model PspB and PspC as a complex, we assume that the same number of both mRNAs is produced; we take this number to be 60 (but could have chosen e.g. 40 as well). Moreover, we assume that the protein numbers mimic this ratio. A fraction of PspA proteins forms a complex with *BC *(eqn. 1g), while the other part forms oligomers (*olg*) by binding of 36 PspA molecules into a complex (eqn. 1f). These oligomers act as effectors and re-establish pmf; we model this by repairing the damaged membrane parts (eqn. 1b). When the membrane is not damaged, proteins PspB and PspC change their conformation back into their native state (eqn. 1j).

When building this model we had to make some further assumptions. Once PspA is in the complex with PspB and PspC, it cannot be used anymore as an effector, i.e. PspA is never released from the complex. Only the newly transcribed PspA can form oligomers which act as effectors to repair the membrane. We also assume that there is no threshold level in terms of proportion of the membrane that needs to be damaged in order to pass the signal on, i.e. we simply assume that the signal is stronger if a larger proportion of the membrane is damaged (i.e. when there are more tokens in the *dm *state), and weaker if a lower proportion of membrane is damaged. This is incorporated into the model through marking-dependent rates; for example, the rate of a *BCAF *break-down (eqn. 1i), and the rate of *BCA *conformational change (eqn. 1k) will be proportional to how much of the membrane is damaged. Another assumption, which is in line with experimental evidence, is that the number of PspF proteins and related constructs (the sum of *F*, *TF *and *BCAF*) is constant in cells, and we therefore incorporate this assumption by excluding production and degradation of PspF from the model. However, we do model production and degradation of the other molecular species (eqns. 1l-1p).

**Figure 4:**
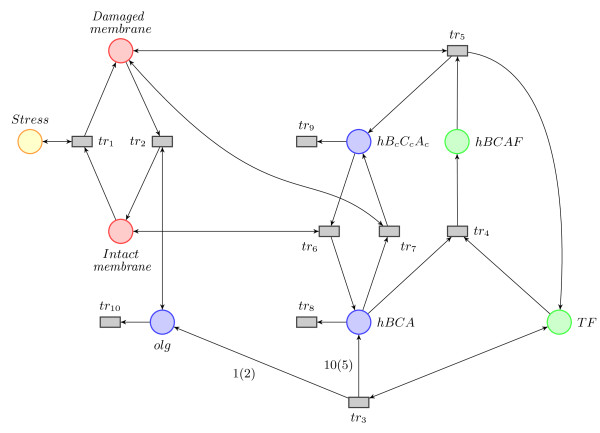
**The simplified Petri net model of the Psp response system**. The simplified Petri net model of the Psp response system. The colour code is as in Figure 3.

The model can be concisely presented as a graphical model in Figure 3 in terms of the following reactions,

We next explore how this model can be simplified further. Since we are only interested in the time course dynamics and want to avoid a large number of unknown parameters, we can remove some of the species and reactions from the model, while still capturing the crucial components of the stress response. As a first simplification step, we model *BCAF*, *B_c_C_c_A_c _*and *BCA *complexes in groups of six (to simplify the hexamer formation of PspF). In a further simplification step we no longer model the production of *A*, *BC *and the subsequent formation of complex *BCA *and oligomers independently (eqns. 1e-1g), but instead model the production of oligomers and *BCA *directly (*tr*_3_, eqn. 2c). The simplified Petri net is now as follows (see Figure 4 for graphic representation),

To complete the definition of a Petri net, we need to define the initial markings. This has to be done with care, as a badly chosen initial marking can result in so called "deadlocks", i.e. when none of the transitions can be fired anymore. A transition is said to be "dead" if it can never fire in any firing sequence. A property related to the absence of deadlocks is liveness, and different levels of liveness exist [18]. A Petri net is L1-live if all transitions can be red at least once in some firing sequence. This property is, for example, satisfied by the following initial marking: *M*_0 _= (*stress; dm, im, olg, hBCA, hB_c_C_c_A_c_, hBCAF, TF*) = (1, 0, 100, 0, 0, 0, 20, 0). That is, we start with the stress turned on, the whole membrane in the intact state, and all Psp proteins present in the system bound in the complex *hBCAF*. There are no oligomers, *hBCA *or *hB_c_C_c_A_c _*complexes in the system, and no transcription factors *TF *available at the start of the simulation. The possible markings are: *stress *∈ {0, 1}; *dm; im *∈ {1, 2, ..., 100}, *im *= 1 - *dm*; *olg*, *hBCA*, ; *hBCAF*, *TF *∈ {1, ... 20}. The marking of a place *dm *can be interpreted as the percentage of membrane damage.

The above reaction scheme can also be transformed into an ODE model [19,20]. This can be done by assuming e.g. mass action kinetics acting on all molecular species. Variables *dm *and *im *are the only non-molecular variables; to de ne an ODE for them we assume a constant rate of change from an intact to a damaged membrane when stress conditions prevail, and the rate of membrane repair to be proportional to the number of oligomers in the system. The ODE model can be written as follows:

with (*y*_1_, *y*_2_, *y*_3_, *y*_4_, *y*_5_, *y*_6_, *y*_7_, *y*_8_) = (*stress, dm, im, olg, hBCA, hB_c_C_c_A_c_, hBCAF, TF *), *y*_1 _∈ {0, 1} and the initial condition *y *= (1, 0, 100, 0, 0, 0, 20*α*, 0), , and  is an indicator function.

Petri net markings form a discrete space (i.e. modelling the numbers of the molecules), while the ODE model variables *y_i_*, *i *= 4, ..., 8 represent concentrations of molecules. Variables *y*_1_, *y*_2 _and *y*_3 _are exceptions in that they do not represent molecules but the stress conditions, *y*_1 _∈ {0, 1} and the state of the membrane, 0 ≤ *y*_2 _≤100, *y*_3 _= 100 - *y*_2_. The relationship between the number of molecules in the Petri net and the concentrations in the ODE model is the following: for a concentration *y_i _*(units *M*/*l*) in a volume of V litres, there are *M_i _*= *n_A_y_i_V *molecules, where *n_A _*≈ 6.02 × 10^23 ^is Avogadro's constant, which represents the number of molecules in a mole [21].

To very good approximation we set the volume of *E. coli *to be 1 *μm*^3 ^= 10^-15^*l *[22]. The number of *M_i _*molecules then corresponds to the concentration of(3)

measured in moles per litre. This applies to the molecular species *olg*, *hBCA*, *hB_c_C_c_A_c_*, *hBCAF*, *TF*.

This conversion rule does obviously not apply to the stress condition, which is either on or off (i.e. 1 or 0), and to the percentages of damaged and intact membrane. For the first order reactions (i.e. of type *X *→ *Y*) the relationship between the stochastic rate *c *and the deterministic rate *k *is *c *= *k*. In our Petri net this rule applies for transitions *tr*_2_, *tr*_3 _and *tr*_5 _- *tr*_10_. For the second order reactions (i.e. of type *X *+ *Y *→ *Z*) this relationship becomes , which applies to transition *tr*_4_, and a zeroth order reaction's (i.e. of type, ∅ → *X*) stochastic rate is *c *= *kn_A_V *, which we use for transition *tr*_1_.

### Model validation and calibration

Employing Petri net terminology we have developed a simple mechanistic model, which summarizes our current knowledge of the phage shock protein response system [8]. We now combine discrete Petri net structural analysis, and stochastic and deterministic simulation and analysis of the model [19]. The classical discrete Petri net theory offers several theoretical tools to analyse structural properties of the Petri net, which are useful for qualitative validation of the model. To validate the basic model structure, we calculate the structural invariants (we explain the meaning of these variants later) and calibrate the dynamic model against qualitative data. This fitting process also provides us with parameter estimates.

In order to obtain the invariants of the Petri net, we can calculate the null space of the reaction matrix

and its transpose (see Methods section for definitions of these terms). A *P-invariant *is a non-zero vector *y *that solves *Ay *= 0, and a *T-invariant *is a non-zero, non-negative vector, *x*, that solves *A^T ^x *= 0.

P-invariants correspond to conservation laws of the network, while T-invariants represent the sequence of transitions that lead back to the initial marking [21]. P- and T-invariants can be used to check the model for consistency, and to test the basic correctness of its biological interpretation [23].

We use the Matlab toolbox for Petri nets [24] to calculate the minimal P- and T-invariants using the algorithm of Martinez and Silva [25]. The P-invariants for our model are given in Table 1. These invariants tell us that the numbers of tokens in *stress, dm + im *and *hBCAF *+ *TF *are constant, which we reflect by the colour scheme in Figure 4. Furthermore, we see that the net is not covered in P-invariants, meaning that the net is in principle unbounded (species *hBCA*, *hB_c_C_c_A_c _*and *olg *do not have an upper bound). In practice, i.e. for finitely lived prokaryotic cells this does not matter, and, as we will show below can be elegantly addressed in the ABC framework.

**Table 1:** P-invariants of the simplified Petri net Psp model

*Stress*	*dm*	*im*	*olg*	*hBCA*	*hB_c_C_c_A_c_*	*hBCAF*	*TF*
1	0	0	0	0	0	0	0
0	1	1	0	0	0	0	0
0	0	0	0	0	0	1	1

The T-invariants are given in Table 2. Starting from some marking *M *and ring the listed reactions will bring the Petri net marking back to its original marking *M*. The biological interpretation of minimal T-invariants that we have obtained is

**Table 2:** T-invariants of the simplified Petri net Psp model

*tr*_1_	*tr*_2_	*tr*_3_	*tr*_4_	*tr*_5_	*tr*_6_	*tr*_7_	*tr*_8_	*tr*_9_	*tr*_10_
1	1	0	0	0	0	0	0	0	0
0	0	0	0	0	1	1	0	0	0
0	0	1	0	0	0	0	10	0	1
0	0	0	1	1	1	0	0	0	0
0	0	1	0	0	0	10	0	10	1
0	0	1	10	10	0	0	0	10	1

• the membrane gets damaged and then repaired; (*tr*_1_, *tr*_2_).

• proteins PspB and PspC change conformation, and then return to back to the original state, (*tr*_6_, *tr*_7_).

• transcription and translation of new PspA, PspB and PspC proteins and their complexes, and their subsequent degradation; (*tr*_3_, 10 *tr*_8_, *tr*_10_), (*tr*_3_, 10 *tr*_7_, 10 *tr*_9_, *tr*_10_).

• binding of protein PspF to the complex of PspA, PspB and PspC, and subsequent breakup of the complex; (*tr*_4_, *tr*_5_, *tr*_6_).

• transcription and translation of new PspA, PspB and PspC proteins, formation of a complex between PspA, PspB, PspC and PspF proteins, the breakup of this complex and protein degradation; (*tr*_3_, 10 *tr*_4_, 10 *tr*_5_, 10 *tr*_9_, *tr*_10_).

All these invariants are biologically sound (and may also be deduced by inspection of the model). While the basic system behaviour is determined by the minimal T-invariants, the linear combinations of these invariants describe all possible behaviours of the system. The results here agree well with the P and T-invariants of the full model in Figure 3, which are given in [Additional file 1].

Having obtained some level of support for the model structure, we next study its dynamics. We are particularly interested in the dynamics after the induction of stress, as well as the dynamics following the subsequent removal of stress (which is experimentally challenging). Despite the fact that many aspects and the molecular players involved in the Psp system have been studied in detail, not much is known about its temporal behaviour. We know that upon the induction of stress, most of the Psp protein levels rise and that the complex between PspA and PspF (*hBCAF *in our model) is likely to be broken down. However, the time course dynamics or kinetic rates (e.g. production and degradation rates) have so far not been measured. Moreover, the effects of removal of stress after stress induction has also never been experimentally studied. Our network model allows us to theoretically predict the possible dynamic behaviour.

We are going to employ stochastic and deterministic simulation and approximate Bayesian computation (ABC) (see Methods) in order to explore what dynamics we can infer from the *qualitative end-point data*. By qualitative end-point data we mean, for example, that at the end of the stress induction period *t*_1 _we expect all the complexes to be broken down (*hBCAF *(*t*_1_) = 0), while at the end of the stress-free period *t*_2_, after the system has had time to recover, we expect all PspF proteins to be bound in the *hBCAF *complex and no free transcription factor to be present (*TF*(*t*_2_) = 0). Since no quantitative data are available, we can rescale all units in terms of the (arbitrary) time scale, and we simulate the dynamics over 40 time units. The stress will be induced during time interval [0, 10), turned off (i.e. removed, washed away) in time interval [10, 30) and induced again in time interval [30, 40). These time intervals have been chosen arbitrarily and we later explore the dependence on the choice of (relative) lengths. The qualitative data can then be cast in the following terms,

where *a *represents the percentage of damaged membrane at the end of the stress induction period. Here we study the behaviour of the system for different values of *a*.

**Figure 5:**
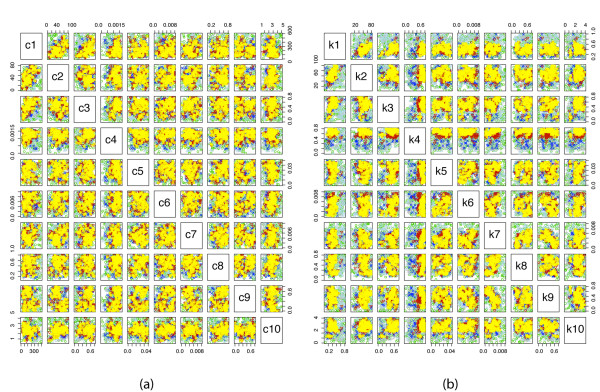
**Parameter scatterplots for stochastic and deterministic Psp models**. Inferred parameter distributions. Shown are the two-dimensional projections of the 10-dimensional intermediate and posterior parameter distributions, i.e. the output of the ABC SMC algorithm consisting of all accepted particles (i.e. parameter combinations). Circles correspond to accepted particles (*k*_1_, ..., *k*_10_), which result in a good fit to the data (see Figure 6). Eight ABC SMC populations were run, and particles from each population are coloured by a different colour. The particles of the last population are coloured in yellow - this population of particles approximates posterior parameter distribution, and its particles are parameter combinations that give the best fit of the model to the data (in a Bayesian sense). The parameter determining the damaged membrane was set to *a *= 60. (a) Parameters inferred in a stochastic frameworks. (b) Parameters inferred in a deterministic framework. The parameters in deterministic framework were sampled from the following priors: *k*_1_, *k*_3_, *k*_4_, *k*_8_, *k*_9 _~ *U *(0, 1), *k*_2 _~ *U*(0, 100), *k*_5 _~ *U*(0, 0.05), *k*_6_, *k*_7 _~ *U*(0, 0.01), *k*_10 _~ U(0, 5). In the stochastic framework, corresponding priors were calculated as explained in section. Tolerance levels used in ABC SMC algorithm: *ε*_1 _= (100, 13.0, 100.0, 100.0, 1.5), *ε*_2 _= (80, 10.0, 100.0, 100.0, 1.3), *ε*_3 _= (60, 8.0, 70.0, 70.0, 1.2), *ε*_4 _= (50, 7.0, 60.0, 60.0, 1.1), *ε*_5 _= (40, 6.0, 50.0, 50.0, 1.0), *ε*_6 _= (30, 5.0, 40.0, 40.0, 0.9), *ε*_7 _= (20, 4.0, 30.0, 30.0, 0.8), *ε*_8 _= (10, 3.0, 20.0, 20.0, 0.7). These tolerance levels together with the distance function (*d*_1_, ..., *d*_5_) defined in the text, determine which proposed particles will be accepted.

**Figure 6:**
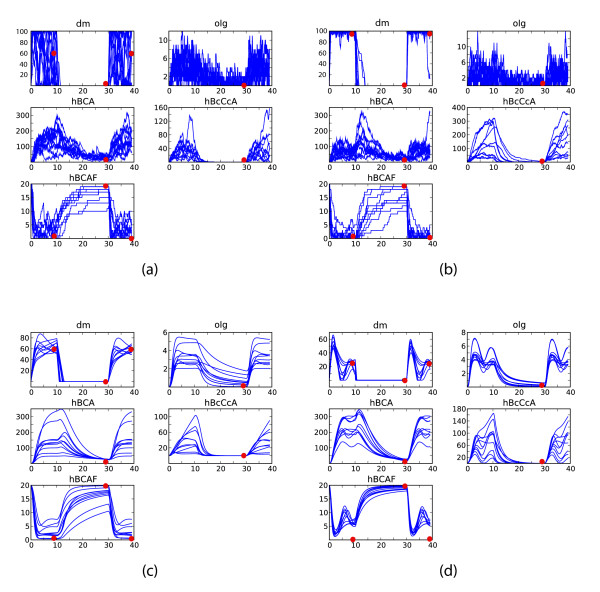
**Psp stochastic and deterministic model fits to the data**. Simulated trajectories fitted to the data. Ten parameter combinations from the inferred approximate Bayesian posterior parameter distribution (Figure 5) were randomly selected and models simulated. The red circles represent the known data. (a)-(b) Stochastic trajectories fitted to "damaged membrane" data points chosen as *a *= 60 and *a *= 10, respectively. (c)-(d) Deterministic trajectories of the ODE model, *a *= 60 and *a *= 25, respectively.

In order to fit the model to the data we use a slight modification to a previously published ABC SMC algorithm (see Methods). For the stochastic simulations we use Gillespie's algorithm, and a numeric ODE solver (odeint in Scipy) for the deterministic simulations; both are implemented in the ABC-SysBio software [26]. We define the distance function as a vector of five functions, on the summary statistics defined above:

As opposed to the previous applications of ABC to dynamical systems [5,27], where the distance was generally chosen to be the sum of squared errors, and where we defined one tolerance level in each population, we now need to define a vector containing five tolerance levels corresponding to the above distance functions for each population. The use of this ABC procedure also allows us to control the potentially unbounded nature of the underlying mathematical model in order to home in onto biologically plausible scenarios for the ODE and stochastic implementations. By inferring the parameters (shown in Figure 5), we constrain the model simulations to realistic behaviours and finite species concentrations (example trajectories simulated with parameters drawn from the posteriors are shown in Figure 6).

Figures 5(a)-(b) show the inferred posterior distributions of the parameters. Illustrated are the two dimensional projections. Reassuringly, posterior distributions of both deterministic and stochastic rates have the same shape, with stochastic parameters allowing a slightly broader range. We can see, for example, that parameter *k*_4 _is already easily inferred from the available qualitative data. Moreover, some parameters are much more restricted (i.e. better inferred) in the deterministic case than in the stochastic case (e.g. *k*_2_), while the other parameters are equally inferable in both cases (e.g. *k*_10_).

Having obtained the posterior parameter distributions, we can now simulate possible dynamic behaviours for different parameter realizations in order to make predictions of the dynamic model output. Figures 6(a)-(b) illustrate the possible stochastic behaviour and Figures 6(c)-(d) the possible deterministic behaviour for randomly chosen parameters. These parameters were sampled from the inferred posterior distributions obtained above by using ABC SMC for calibrating the model against the end-point data, represented by red dots. We present the results for different proportions of the damaged membrane *a*.

The trajectories generated from our posterior distribution over the model parameters do indeed provide interesting insights into the dynamics of membrane damage (*dm*). The proportion of damaged membrane is an indicator of the severity of the induced stress. We investigate the dynamics in response to different stress severity (*a *ranging from 15 up to 100, results shown for some representative values only). Under deterministic dynamics and when the damage is expected to be low (i.e. low *a*), oscillations may be observed (Figure 6(d)) for many parameters. This behaviour can be explained by the quick initial response to stress, which is then counteracted and attenuated by the membrane repair. The response machinery (specifically, membrane repair through PspA oligomers) acts as a negative feedback on stress induction. On the other hand, if the stress is strong (i.e. high *a*), then the repair machinery will have a smaller effect on the membrane relative to the damage induced by stress (Figure 6(c)). The lower the signal, the more pronounced the oscillations will be in molecular species *olg*, *hBCA*, *hB_c_C_c_A_c_*, *hBCAF *and *TF *as well.

When the stochastic framework is employed (Figures 6(a)-(b)), the membrane damage fluctuates a lot (i.e. from nearly completely damaged membrane to almost intact membrane) and rapidly. But this is again less pronounced when the stress is strong (Figure 6(b)). Another interesting feature that we can observe from the simulated stochastic trajectories is the pronounced difference in the noise levels of different protein complexes. The highest variation is present in *olg*, followed by *hBCA *and *hBCAF*. Interestingly, *hB_c_C_c_A_c _*exhibits relatively low noise; presumably this is due to its frequency being a function primarily of the stress induction and is only very indirectly influenced by other processes.

**Figure 7:**
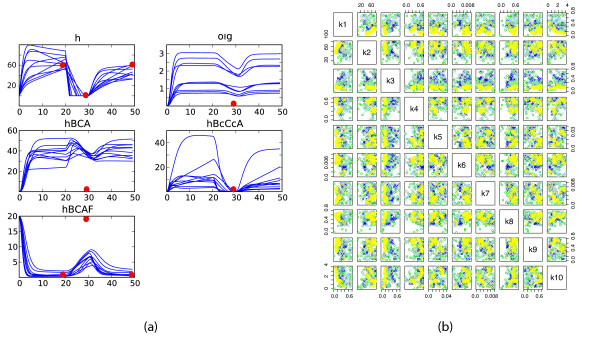
**Psp inference results for a different stress stimulation schedule**. Repeated model fitting and parameter inference for *a *= 60 and a different stress stimulation schedule: stress turned on in [0, 20) and [30, 50), and turned off in [20, 30). (a) Simulated trajectories of the ODE model fitted to the data. (b) Scatterplots of inferred posterior parameter distributions.

In the above analysis we have chosen arbitrary time intervals of stress induction and removal. We therefore repeat the parameter inference procedure for a different stress induction schedule: stress is turned on during intervals [0, 20) and [30, 50), while it is removed from the system in [20, 30). The results are presented in Figures 7(a)-(b). Two features are noticeable from the obtained results. First, the fits are not as good as for the previous stress stimulation schedule (Figure 6(c)), and second, the inferred parameter distributions are different, which can be seen by comparing Figures 5(b) and 7(b). These suggest that the chosen prior ranges do not allow for a quick adaptation to a normal state during a very short stress removal period. The overall qualitative behaviour of the system is, however, in good agreement with the results outlined above.

The next step for modelling the Psp response must be obtaining real experimental quantitative and time-resolved measurements. These will allow for the improved estimation of posterior parameter distributions, and by having confidence in parameter estimates inferred from quantitative experimental data we can then explore the limits and behaviour of the Psp system response when exposed to different stress induction and removal schedules. In particular, even a small number of additional measurements would allow to determine the extent to which oscillatory behaviour is likely to occur in reality.

## Conclusions

Our study was motivated by the following general questions: Can knowledge about quantitative stress response dynamics be inferred from available qualitative data? And can we thereby generate hypotheses which can be tested experimentally? We have approached these problems in an inference-based manner. This means that we have developed a basic model structure and tested its consistency using tools from Petri net theory; for the proposed structure of our network model we have then shown that we can use an ABC SMC algorithm to identify regions in parameter space that allow the model to reproduce the observed (or desired) qualitative behaviour, and we have applied this framework to the Phage shock protein stress response system in *E. coli*. From the resulting posterior distributions over model parameters we were then able to sample plausible model parameters (in the sense that they are in concordance with our present state of knowledge about the system's behaviour) to study the type of deterministic and stochastic dynamical behaviour likely to arise for the Psp response.

The Psp system is part of the sophisticated stress response machinery that *E. coli *has acquired over the course of evolution in order to respond to adverse environmental conditions [4]. The intricate interplay between the different constituent components of the Psp reponse, like many other signal transduction systems, has only been studied in a traditional reductionist approach where the focus is on individual proteins, their structure and their interactions. Although these studies have already provided important insights into the stress response, there is a need for consolidating these (sometimes somewhat disparate) pieces of information into a mechanistic model of the stress response system. Here we have developed an inferential framework to analyze such models quantitatively in light of the available qualitative data.

We have shown that for the Psp response system the limited qualitative and semi-quantitative data alone can already provide some insight into the dynamic nature of the stress response. We have been able to narrow down the parameter regions (i.e. we obtained the posterior parameter distributions in a Bayesian sense) for deterministic and stochastic dynamics of the Psp system. Furthermore, we have predicted the possible dynamic behaviour for all the molecular species involved in the response; furthermore, analysis of the stochastic dynamics has allowed us to predict the relative levels of noise in all of the molecular species. Most importantly, the predicted dynamic behaviour shows a non-trivial and *a priori *unexpected dependence on the stress intensity; oscillations can be observed for low stress intensity for many parameter values that are in agreement with present data, while no oscillations are observed for high stress intensities. Such oscillations could underly population heterogeneity and help to drive differences between responses of otherwise identical cells to environmental stresses. This in turn has recently been shown to have important implications to e.g. drug treatment and escape of some cells from therapeutic interventions [28].

The next step will be to collect quantitative time course data, including the basal level expression of *psp *genes, and "titrate" stress. Advances in quantitative real time live cell imaging methods applied to visualising the psp response across a range of stress conditions, magnitudes and durations of applied stresses are expected to yield the key data needed to examine oscillatory behaviour. These methods produce highly resolved data that will also enable us to target directly the role and biological relevance of oscillatory behaviour of the Psp response system.

This analysis has provided predictions of possible qualitative time course dynamic behaviours of crucial players in stress response. Our model of the Psp system has necessarily (given the amount of available data) focused on the core of this stress response. It would be desirable to extend the model by adding further layers of detail and separate the PspB and PspC proteins into two separate variables, since the proteins are passing the signal on independently; conformational change of PspC is a result of mechanical changes in the membrane, while PspB changes its conformation as a result of chemical changes, and activates the phosphorylation of ArcB and hence ArcA. It is believed that ArcA plays a role in amplification of the signal [13], and it would be of interest to incorporate ArcA explicitly into the model and study how such amplifications are mediated in practice. The ArcA/ArcB two-component system is also involved in other responses to environmental stimuli and is a potential relay of cross talk into the Psp response; capturing the effects of cross-talk will almost certainly require more involved mathematical formalism in order to understand the different contributing factors [29].

We initially developed and applied ABC SMC to deterministic and stochastic dynamical systems as a means of quantitative inference from quantitative time series data [5,30]. In this paper we have applied ABC SMC in a slightly different context and have shown that it can successfully be applied to different and more limited types of data. Another difference to previous applications is that here our main purpose was not to infer parameters, but mainly to explore the likely range of qualitatively different trajectories that could reproduce the data. The scope for this strategy is considerable: it can be applied across all simulation models, and can perform inference tasks from limited, qualitative or quantitative data.

One such area of potentially fruitful application is in the comparative analysis of biological systems. For example, it is well known that some bacterial species, some of which are evolutionary closely related to *E. coli*, lack certain *psp *genes [31]. An adaptation of our current Psp model could then be used to study the likely changes in stress response dynamics by removing these genes from the model. This will allow us to predict how the dynamic stress response in species lacking specific molecular players differs from the stress response of the well studied model organism *E. coli*.

To take this approach one step further still, one can propose a set of candidate models and fit them to data representing the desired behaviour of the system. Then a model selection approach [30] can be employed in order to determine which of the proposed models reproduces the desired behaviour most reliably and most robustly. Such an approach can for example be used to guide the design of synthetic biological systems [32]; the function or action that the synthetic system is required to perform can be described by qualitative data (e.g. oscillations or production of a specific protein etc.) and candidate models can be fitted to these data (which are really design objectives) using an appropriate model selection technique in order to (i) choose which model will best reproduce the behaviour we would like the system to undertake, and (ii) infer the parameter distributions. In simulation-based studies we have found this to be a very promising and intuitive strategy to come up with signal transduction pathways that respond to stimuli in the environment in a desired and specified manner.

## Methods

### Introduction to Petri nets

Petri nets [33] are a graphical and mathematical modelling tool applicable to many systems and are often used to study concurrent processes [18]. Different kinds of Petri nets have been developed and applied for modelling biological pathways including metabolic pathways, gene regulatory networks, signal transduction pathways and integrated signalling-regulatory systems. Reviews and extensive bibliographies can be found in references [15-17,34]. Here the main purpose of using them is as a convenient pictorial representations that allows for the efficient exchange of ideas between biological domain experts and modellers.

**Figure 8:**
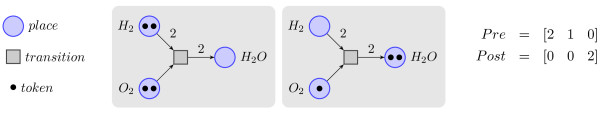
**An example Petri net**. Petri net representation of a chemical reaction, 2*H*_2 _+ *O*_2 _→ 2*H*_2_*O*. The rows in *Pre *and *Post *matrices correspond to the Petri net transitions (in this example there is only one transition) and the columns to the three places in the following order: *p*_1 _= *H*_2_, *p*_2 _= *O*_2 _and *p*_3 _= *H*_2_*O*. If no weight is written on the arc, this corresponds to weight 1. The initial marking is *M*_0 _= [2,2,0]^*T *^and after the reaction has been red the marking becomes *M *= [0,1,2]^*T*^.

In simple and descriptive terms, a Petri net is a graphical model that consists of places, transitions and arcs (a simple example is given in Figure 8). Petri nets are well suited for describing temporal dynamics: when a transition is fired, tokens are moved between places. Simulation in combination with the rich analytic theory for studying Petri nets have proven useful in helping us to understand the behaviour of complex systems.

In the following paragraphs we de ne the components of a Petri net and give a biological interpretation following Goss and Peccoud [35]. A Petri net is a directed bipartite graph, in which directed arcs connect two types of nodes: places *P *= {*p*_1_, ..., *p_n_*} and transitions *T *= {*tr*_1_, ..., *tr_m_*}. In a graphical representation, circles represent places and rectangles represent transitions. To model a system of molecular interactions as a Petri net, each place represents a distinct molecular species or condition. These places contain tokens, which represent individual molecules or other biological entities. The number of tokens in a place, *p_i_*, is its marking, and the state of all places is called a global marking, *M*. The initial marking, *M*_0_, represent the number of tokens in each place at time *t *= 0.

Transitions represent chemical reactions or a change from one molecular state to another. Directed arcs, which represent input and output functions, link places to transitions and transitions to places. Each arc has an associated weight;  (where  are the non-negative integers) is the matrix containing the weights of arcs going from places to transitions, and  contains the weights of arcs going from transitions to places. These weights determine the stoichiometric coefficients of the species involved in the reaction. A transition *tr_i _*is said to be enabled when the marking of a place is equal to or greater than the coefficient of its corresponding input arc, *M_j _*≥ *Pre_ij_*, *j *= 1, ..., *n*. Enabled transitions can "fire", moving the tokens from input to output places. This defines the place/transiton Petri net.

However, if we want to study how molecular species change in time, we need to incorporate a time component into a Petri net. Petri nets in which transitions fire in discrete time (e.g. *t*, *t *+ 1, *t *+ 2, . . .) are called timed Petri nets. Molecular events, however, are known to be governed by stochastic rate laws, which can be modelled by a Stochastic Petri net [36]. A Stochastic Petri net is derived from a place/transition Petri net, by assigning the rates to transitions; these rates are marking dependent and in the present context the markings of the stochastic Petri net are discrete and represent the number of molecular species. The times at which transitions fire are exponentially distributed and given the kinetic laws the stochastic Petri net can be simulated using Gillespie's algorithm [37].

If the number of molecules is sufficiently large and stochastic fluctuations can be ignored then we can choose to study how concentrations, rather than numbers of molecules, changer over time. We therefore further transform the Petri net into a timed continuous model, which can also be described with ordinary differential equations (how this is done, is described in detail elsewhere [19,20]). Here, a continuous Petri net is just a convenient graphical representation of a dynamical system, which for modelling purposes is often described by deterministic, ordinary differential equations.

Modelling of chemical reactions, such as the one in the above example, is quite straightforward as the basic purpose of Petri nets is to represent production/consumption processes. In the same light, metabolic networks, which consist of biochemical reactions, can naturally be represented by a Petri net. However, genetic regulatory networks are more difficult to model with Petri nets. While the reactants get consumed in the metabolic networks, the regulators do not turn over during a regulatory process: in addition to flux of matter, the flow of information gains in importance. Therefore, a slightly different Petri net structure is needed for modelling regulatory networks. The situation is similar in signal transduction networks; molecules *respond *to signals rather than turn over. For example, a molecule might (transiently) change conformation in response to a signal. Another example is the binding of a transcription factor to DNA that result in new proteins being produced (without consumption of transcription factor). This type of information flow can be modelled by test arcs. Whenever a test arc is used in our model, the number of tokens in the "test place" does not change, and the rate of transition is dependent on the number of tokens (i.e. marking-dependent). The test places in our model are *stress, dm, im, TF *and *olg*.

In this manuscript, Petri nets are used in the following way: the most basic, place/transition Petri nets are used to validate the basic structure of the model and determine the initial markings. Petri nets, which define the structure of our model, are then turned into stochastic and continuous (deterministic) simulation frameworks, which are used to study the dynamics.

### Approximate Bayesian computation (ABC)

ABC methods have been developed in order to obtain Bayesian posterior distributions where likelihood functions are computationally intractable or too costly to evaluate [5]. They replace evaluation of the likelihood with a comparison of observed and simulated data. Let *θ *be such a parameter vector to be estimated. Given a suitable prior distribution, *P*(*θ*), our goal is to develop an approximation to the posterior, *P *(*θ *| *D*_0_) ∝ *f *(*D*_0 _| *θ *)*P*(*θ*), where *f *(*D*_0 _*|θ*) is the likelihood of *θ *given the data *D*_0_. ABC methods take the following generic form:

**1 **Sample a candidate parameter vector θ* from a suitable proposal distribution *P*(*θ*) (our main constraint is that *P*(*θ*) > 0 wherever we expect the posterior to have weight).

**2 **Generate a simulated dataset D* from the conditional probability distribution *f *(D|θ*).

**3 **Compare simulated and experimental data sets, D* and *D*_0_, respectively, using a distance function, *d*, and tolerance *ε*; if *d*(*D*_0_, D*) ≤ *ε*, accept θ*, otherwise reject θ* and return to **1**. Here *ε *≥ 0 is the required level of agreement between *D*_0 _and D*.

The output of an ABC algorithm is a sample of parameters from the distribution

which for sufficiently small *ε *is our approximation for the true posterior distribution, P(*θ*|*D*_0_). Instead of defining a distance function *d*(*D*_0_, D*) between the full datasets, it may be more convenient to define it on sufficient summary statistics, *S*(*D*_0_) and *S*(D*), of the datasets. That is, the distance function may be defined as *d*(*D*_0_, D*) = *d*'(*S*(*D*_0_), *S*(D*)), where *d*' is a distance function on the summary statistic space.

The simplest ABC algorithm is the ABC *rejection sampler *[38], which repeatedly executes the generic ABC building block presented above. The disadvantage of the ABC rejection sampler is that the acceptance rate is low when the prior distribution is very different from the posterior distribution, and this will nearly always be the case in real-world applications. In order to speed up the procedure, ABC SMC has been developed [5,39,40].

### ABC based on sequential Monte Carlo (SMC)

In ABC SMC particles, {*θ*^(1)^, ..., *θ*^(*N*)^}, sampled from the prior distribution, *P*(*θ*), are propagated through a set of intermediate distributions, *π*(*θ*|*d*(*D*_0_, D*) ≤ *ε_i_*), *i *= 1, ..., *T *- 1, until it corresponds to a sample from the target distribution, *π*(*θ*|*d*(*D*_0_, D*) ≤ *ε_T_*). The tolerance schedule, *ε_i_*, is chosen such that *ε*_1 _> . . . >*ε_T _*≥ 0; thus the distributions gradually evolve towards the target posterior. The *ABC SMC algorithm *proceeds as follows:

**S1 **Initialize *ε*_1_, ..., *ε_T _*and set the population indicator *t *= 1.

**S2.0 **Set the particle index *i *= 1.

**S2.1 **When *t *= 1 sample θ** directly from *P*(*θ*).

Otherwise sample θ* from  with weights *w_t _*_- __1 _and perturb sampled particles to obtain θ** ~ *K_t_*(θ|θ*), where *K_t _*is the perturbation kernel.

When *P *(θ**) = 0, return to **S2.1**.

Generate a simulated dataset D* ~ *f *(*D*| θ**).

If *d*(*D*_0_, D*) ≥ *ε_t_*, return to **S2.1**.

**S2.2 **Set  and determine the weight corresponding to  as

If *i *<*N *set *i *= *i *+ 1, go to **S2.1**.

**S3 **Normalize the weights.

If *t *<*T*, set *t *= *t *+ 1 and return to **S2.0**.

Perturbation kernels *K_t _*are chosen here to be random walk (uniform or Gaussian) processes, but other choices are possible; in principle any update (e.g. from genetic algorithms) can be used as long as weights can be calculated. For a "friendly" introduction to ABC SMC we refer to [41].

This algorithm requires the provision of suitable prior distributions, distance functions, tolerance schedules and perturbation kernels. We choose uniform prior distributions for all parameters; this still allows us to constrain parameters -- e.g. such that all reaction rates are positive and within physiological ranges -- but otherwise makes no extraneous assumptions about the system. Tolerance schedules need to be defined empirically to speed up convergence. The perturbation kernels we employ are uniform and are automatically adapted for population *t *by feeding it information on the obtained parameter ranges from population *t *- 1:(4)

where *σ_t _*depends on the length of a parameter range achieved in population *t *- 1, e.g.(5)

This choice of kernel ensures good mixing and has been found to capture the extent of the posterior distribution faithfully; for kernels with narrower bandwitdh (due to the finite number of particles as well as the hard rejection criterion in **S2.1 **above) the variance of the posterior is likely to be underestimated [5,40].

## Authors' contributions

TT and MPHS designed the research and wrote this manuscript. TT, GJ, MH, MB and MPHS developed the model. TT analyzed the model. All authors have read and approved the manuscript.

## Supplementary Material

Additional file 1 **Petri Net Invariants for the Full Model**. Table 1 and Table 2 show the P and T invariants of the full Petri net model shown in Figure 3. Click here for file
