# Supplementary material for: From qualitative data to quantitative models: analysis of the phage shock protein stress response in Escherichia coli
Source: BMC Syst Biol. 2011 May 12;5:69. doi: 10.1186/1752-0509-5-69 (PMC3127791; doi:10.1186/1752-0509-5-69)
Supplement: Additional file 1 — Petri Net Invariants for the Full Model. Table 1 and Table 2 show the P and T invariants of the full Petri net model shown in Figure 3. [file 1752-0509-5-69-S1.PDF]

Supplementary material

Table 1 - P-invariants of the starting Petri net Psp model

$stress$	dm	im	F	TF	A	BC	BCA	$B_cC_{C_c}A_c$	olg	$BCAF$
1	0	0	0	0	0	0	0	0	0	0
0	1	1	0	0	0	0	0	0	0	0
0	0	0	1	6	0	0	0	0	0	1

Table 2 - T-invariants of the starting Petri net Psp model

tr_1	tr_2	tr_3	tr_4	tr_5	tr_6	tr_7	tr_8	tr_9	tr_{10}	tr_{11}	tr_{12}	tr_{13}	tr_{14}	tr_{15}	tr_{16}
1	1	0	0	0	0	0	0	0	0	0	0	0	0	0	0
0	0	0	0	1	1	0	0	0	0	0	0	0	0	0	0
0	0	0	1	0	0	0	0	0	0	0	0	0	100	60	0
0	0	25	9	0	0	0	0	0	0	0	0	0	0	540	25
0	0	0	0	0	0	0	1	1	0	0	0	0	0	0	0
0	0	0	0	0	0	0	1	0	1	1	0	0	0	0	0
0	0	0	5	0	0	300	0	0	0	0	0	300	200	0	0
0	0	50	45	0	0	2700	0	0	0	0	0	2700	0	0	50
0	0	0	5	0	0	300	0	300	0	0	300	0	200	0	0
0	0	0	5	0	0	300	0	0	300	300	300	0	200	0	0
0	0	50	45	0	0	2700	0	2700	0	0	2700	0	0	0	50
0	0	50	45	0	0	2700	0	0	2700	2700	2700	0	0	0	50
